# The carotid plaque imaging in acute stroke (CAPIAS) study: protocol and initial baseline data

**DOI:** 10.1186/1471-2377-13-201

**Published:** 2013-12-13

**Authors:** Anna Bayer-Karpinska, Florian Schwarz, Frank A Wollenweber, Holger Poppert, Tobias Boeckh-Behrens, Alexander Becker, Dirk A Clevert, Konstantin Nikolaou, Christian Opherk, Martin Dichgans, Tobias Saam

**Affiliations:** 1 Institute for Stroke and Dementia Research, Klinikum der Universität München, Ludwig-Maximilians-University, Marchioninistr 15, 81377 Munich, Germany; 2 Institute for Clinical Radiology, Klinikum der Universität München, Ludwig-Maximilians-University, Marchioninistr 15, 81377 Munich, Germany; 3 Institute for Clinical Radiology, Klinikum der Universität 555 München, Ludwig-Maximilians-University, Pettenkoferstr 8a, 80336, 556 Munich, Germany; 4 Department of Neurology, Klinikum der Universität München, Ludwig-Maximilians-University, Marchioninistr 15, 81377 Munich, Germany; 5 Interdisciplinary Stroke Center, Klinikum der Universität 559 München, Ludwig-Maximilians-University, Marchioninistr 15, 81377, 560 Munich, Germany; 6 Department of Cardiology, Klinikum der Universität München, Ludwig-Maximilians-University, Marchioninistr 15, 81377 Munich, Germany; 7 Department of Neurology, Klinikum Rechts der Isar, Technische Universität München, Ismaninger Str. 22, 81675 Munich, Germany; 8 Department of Neuroradiology, Klinikum Rechts der Isar, Technische Universität München, Ismaninger Str. 22, 81675 Munich, Germany

**Keywords:** Atherosclerosis, Cryptogenic stroke, Internal carotid artery, Plaque imaging, MRI

## Abstract

**Background:**

In up to 30% of patients with ischemic stroke no definite etiology can be established**.** A significant proportion of cryptogenic stroke cases may be due to non-stenosing atherosclerotic plaques or low grade carotid artery stenosis not fulfilling common criteria for atherothrombotic stroke. The aim of the CAPIAS study is to determine the frequency, characteristics, clinical and radiological long-term consequences of ipsilateral complicated American Heart Association lesion type VI (AHA-LT VI) carotid artery plaques in patients with cryptogenic stroke.

**Methods/Design:**

300 patients (age >49 years) with unilateral DWI-positive lesions in the anterior circulation and non- or moderately stenosing (<70% NASCET) internal carotid artery plaques will be enrolled in the prospective multicenter study CAPIAS. Carotid plaque characteristics will be determined by high-resolution black-blood carotid MRI at baseline and 12 month follow up. Primary outcome is the prevalence of complicated AHA-LT VI plaques in cryptogenic stroke patients ipsilateral to the ischemic stroke compared to the contralateral side and to patients with defined stroke etiology. Secondary outcomes include the association of AHA-LT VI plaques with the recurrence rates of ischemic events up to 36 months, rates of new ischemic lesions on cerebral MRI (including clinically silent lesions) after 12 months and the influence of specific AHA-LT VI plaque features on the progression of atherosclerotic disease burden, on specific infarct patterns, biomarkers and aortic arch plaques.

**Discussion:**

CAPIAS will provide important insights into the role of non-stenosing carotid artery plaques in cryptogenic stroke. The results might have implications for our understanding of stroke mechanism, offer new diagnostic options and provide the basis for the planning of targeted interventional studies.

**Trial Registration:**

NCT01284933

## Background

Ischemic stroke is one of the leading causes of disability and death in industrialized countries [1]. Current etiologic classification systems like TOAST (trial of Org 10172 in Acute Stroke Treatment) [2] or A-S-C-O [3] assign stroke causes to one of four major categories: large artery atherosclerosis (LAA), cardioembolism (CE), small vessel disease (SVD) or stroke of other determined etiology. However, in up to 30% of cases the etiology remains unknown (cryptogenic stroke) even in well characterized patients [4,5]. A review by Amarenco suggested that a significant proportion of these cases may be due to non-stenosing atherosclerotic plaques or low grade cervical artery stenosis not fulfilling common criteria for atherothrombotic stroke [6].

Previous histopathological examinations have demonstrated a role of plaque rupture in myocardial infarction [7] as well as atherosclerotic stroke [8]. Plaque rupture depends on plaque vulnerability rather than plaque size. Plaques with a lipid rich necrotic core, a thin fibrous cap, marked inflammation and intraplaque hemorrhage have been shown to predispose to rupture and thromboembolic complications [9-11]. Despite the obvious association of plaque composition and its vulnerability, current treatment algorithms for carotid artery atherosclerosis use stenosis grade as decision criterion whereas plaque characteristics are usually not considered [12,13].

High-resolution black-blood carotid magnetic resonance imaging (carotid MRI) allows non-invasive characterization of atherosclerotic plaques by the assessment of plaque size, plaque composition, and plaque morphology with good correlation to histopathology [14,15]. Complicated American Heart Association lesion type (AHA-LT) VI plaques are characterized by plaque surface rupture, luminal thrombosis and intra-plaque hemorrhage [14]. Furthermore, progression of atherosclerotic disease burden can be monitored non-invasively [16,17].

Previous MR plaque imaging studies on patients with carotid artery stenosis have revealed an association between the presence of complicated AHA-LT VI features and ischemic events in cross-sectional [18,19] and longitudinal studies [20,21]. However, most of these studies were performed in patients with high grade stenosis [20,21] and mixed cohorts of both asymptomatic and symptomatic patients [18,22]. Furthermore, they used a broader definition of the index event [19], and time between symptoms and carotid plaque MRI was up to 3 months [23]. In a prospective pilot study on 32 cryptogenic stroke patients with non-stenosing carotid artery plaques we recently found a prevalence of 37% for complicated AHA-LT VI plaques ipsilateral to the ischemic stroke compared to the contralateral side, where no AHA-LT VI plaques were found (p = 0.001) [24]. These findings suggest a possible causative role of complicated AHA-LT VI plaques in patients with cryptogenic stroke.

We thus initiated the current prospective CAPIAS study with the primary aim to determine the prevalence of complicated AHA-LT VI plaques ipsilateral to an acute ischemic stroke in patients with a cryptogenic stroke. Secondary outcome measures include the association of complicated AHA-LT VI plaques with specific infarct patterns, biomarkers and aortic arch plaques, the evaluation of recurrence rates of ischemic events up to 36 months and rates of new ischemic lesions on cerebral MRI (including clinically silent lesions) after 12 months as well as the evaluation of the influence of specific AHA-LT VI plaque features on the progression of atherosclerotic disease burden.

## Methods

### Study design

CAPIAS is an observational hospital-based cohort study in patients suffering from acute stroke conducted at three University hospitals in Germany. The study was initiated in February 2011 at the Interdisciplinary Stroke Center in Munich (Ludwig-Maximilians-University) and subsequently extended to the Technical University Munich and the University of Freiburg. The centers use identical core protocols, particularly with regard to the radiological requirements and identical data collection methods using TeleForm (Electric Paper GmbH, Lüneburg, Germany). The study has been approved by each local ethics committee (project 016–10, 20.12.2010), is conducted according to the Declaration of Helsinki and has been registered at the trial registration website clinicaltrials.gov (NCT01284933). Patient insurance for the study was contracted (policy number 40-958405-03029/390). No randomization and study-related interventions are planned. Patients are treated following current guidelines for the treatment of stroke [25].

### Patient population - Inclusion and exclusion criteria

Patients of both genders older than 49 years presenting with neurological symptoms compatible with an acute unilateral anterior circulation infarct within the last seven days are recruited through certified stroke units at the participating centers. DWI positive lesions in the territory of a single internal carotid artery (ICA) on cerebral MRI and the presence of plaques with ≥ 2 mm thickness on one of the carotid arteries as defined by ultrasound are required for inclusion. Patients with high-grade carotid artery stenosis (≥70% NASCET) are excluded from the study as these patients usually undergo immediate invasive treatment (carotid endarterectomy or carotid artery stenting) following current guidelines. Additional exclusion criteria include bilateral ischemic lesions and infarcts within the posterior circulation. For detailed inclusion and exclusion criteria see Table 1.

**Table 1:** Inclusion and exclusion criteria

**Inclusion criteria**	**Exclusion criteria**
• Age > 49 years old	• Primary referral to an outside hospital
• Acute ischemic stroke or transient ischemic attack (TIA)	• DWI positive lesions outside the territory of a single internal carotid artery
• Neurological symptoms compatible with a stroke or TIA in the territory of the internal carotid artery	• Surgical procedure within 24 hours preceding the MRI
• Onset of symptoms within the last 7 days	• History of radiation to the neck area
• 1 or more acute ischemic lesion(s) visible on MR diffusion-weighted imaging (DWI) in the territory of a single internal carotid artery	• Carotid artery stenosis ≥70% (NASCET) ipsilateral to the stroke or TIA as defined by ultrasound (systolic peak flow velocity ≥ 300 cm/s)
• Presence of carotid artery plaques in the ipsi- or contra-lateral carotid artery as defined by ultrasound (plaque thickness at least 2 mm; located within 1 cm proximal or distal to the carotid bifurcation)	• Creatinine levels > 2 times the upper limit of the standard range of the respective laboratory within the last 30 days prior to MRI and Renal clearance < 30 ml/minute
• Written informed consent	• Standard contra-indications for MRI

In our study “Cryptogenic stroke” is defined as a stroke `of undetermined origin’ according to TOAST classification, which is equivalent to the absence of grade 1 pathology according to A-S-C-O. Thus, patients with concurrent etiologies will be analyzed as a separate group. Patients with cardioembolic stroke or small vessel disease represent the negative control group and patients with large artery atherosclerosis (50-69% stenosis) serve as positive control.

### Primary outcome

The primary outcome of the CAPIAS study is the prevalence of complicated AHA-LT VI plaques ipsilateral to an acute ischemic stroke in the territory of a single carotid artery in patients with a cryptogenic stroke. The analyses will include a comparison of the prevalence of complicated AHA-LT VI plaques ipsilateral versus contralateral to the ischemic stroke and a comparison of complicated AHA-LT VI plaques in patients with cryptogenic stroke as compared to patients with defined stroke etiology.

### Secondary outcomes

Secondary outcomes include the association of complicated AHA-LT VI plaques with specific infarct patterns (territorial infarcts, multiple embolic infarcts, small lacunar lesions), with aortic arch plaques as determined by transoesophageal echocardiography or computed tomography angiography (CTA) and their association with biomarkers derived from serum and plasma. Furthermore, we will evaluate the recurrence rate of ischemic events and the rate of new ischemic lesions on brain MRI in patients with complicated AHA-LT VI plaques after twelve months follow up. In addition, we will investigate whether specific features of complicated AHA-LT VI plaques, such as intraplaque hemorrhage and thin or ruptured fibrous cap are associated with an increased progression rate of atherosclerotic disease burden after twelve months.

Recurrent ischemic events include cerebrovascular and cardiovascular events. Cerebrovascular events are defined either clinically (TIA, amaurosis fugax or stroke), by brain MRI alone (new ischemic lesion on brain MRI) or on the combined assessment of clinical findings and brain MRI. Furthermore, the most likely cause of the recurrent cerebrovascular event is classified according to the ASCO and TOAST classifications. Unilateral events in the anterior circulation will be included in the final analysis to estimate the risk of recurrent events in patients with AHA-LT VI plaques. Recurrent ischemic events in other territories, bilateral events and cardiovascular events (myocardial infarction, vascular death) are recorded during follow-up to evaluate if patients with complicated AHA-LT VI plaques have a higher overall atherosclerotic disease burden.

### Assessment

All patients included into the study are interviewed and examined within 7 days after stroke onset. Assessment at 3, 24 and 36 months follow up is performed using a structured telephone interview conducted centrally by the coordinating center (LMU Munich). Twelve month follow up consists of a clinical examination by a stroke neurologist, blood investigations as well as repeated carotid and brain MRI. Table 2 gives a detailed schedule of planned assessments and time points. In cases where no contact with the patient can be established, information will be obtained from the Residents’ Registration Office.

**Table 2:** Schedule of assessments

**Month**		**0**	**3**	**12**	**24**	**36**
Informed consent		X				
**Interview**						
Demographic variables, migration status		X				
Living situation and level of independence		X	X	X	X	X
Vascular risk factors		X				
Family history (cardiovascular diseases)		X				
Health history (cardiovascular & neurological diseases)		X				
Medication		X	X	X	X	X
Incident cardiovascular & neurological diseases			X	X	X	X
**Neuropsychological Examinations**						
	Montreal Cognitive Assessment (MoCA)	X		X		
	Mini Mental Status Examination (MMSE)	X		X		
	Modified Telephone Interview for Cognitive Status (TICS)		X		X	X
**Clinical Examinations**						
	Anthropometry (Weight, Height, Waist circumference)	X		X		
	Blood pressure	X		X		
	Physical & neurological examination	X		X		
	National Institute of Health Stroke Scale (NIHSS)	X		X		
	Modified Rankin Scale (mRS)	X	X	X	X	X
	Barthel Index (BI)	X	X	X	X	X
**Technical Examinations**						
Blood draws for biobanking, laboratory investigations		X		X		
electrocardiography (ECG)		X		X		
24-hour ECG		X				
Screening ultrasound of the carotid arteries		X				
Transoesophageal and transthoracic echocardiography		X				
Cerebral Magnetic Resonance Imaging (MRI)		X		X		
High resolution black blood carotid MRI (carotid MRI)		X		X		
Contrast enhanced ultrasound (CEUS)		X				

#### Clinical and neuropsychological assessment

Information about demographics, living situation, functional outcome, lifestyle habits, health and family history as well as medication before stroke are provided by the patient or the next of kin. Cardiovascular Risk Factors are defined according to current guidelines [26]. Cognitive function is investigated using the Mini Mental State Examination (MMSE) [27] and the Montreal Cognitive Assessment (MoCA) [28]. The Informant Questionnaire on Cognitive Decline in the Elderly (IQCODE) [29] will be performed in subjects scoring below 27 in the MoCA. Clinical examination consists of anthropometric measures and a standardized neurological examination. Stroke severity is assessed by a certified stroke neurologist using the National Institutes of Health Stroke Survey (NIHSS). The modified Rankin Scale (mRS) and the Barthel Index (BI) serve as measures of functional outcome.

#### *Brain MRI*

Unilateral infarct localization within the anterior circulation is confirmed by brain MRI. Infarcts are defined as acute diffusion restrictions (hyperintensities) on Diffusion weighted images with corresponding hypointense areas on ADC maps. In addition T2-weighted and FLAIR sequences are acquired.

#### *Ultrasound*

Ultrasound investigations include assessment of extra- and intracranial vessels by Doppler and color duplex sonography. Specifically, the following measures are obtained: plaque thickness, plaque localization (< 1 cm proximal or distal to carotid bifurcation), plaque configuration (eccentric or concentric), echogenicity (hypo-, iso- or hyperechoic) and stenosis grade according to NASCET criteria.

#### *Cardiac examinations*

Cardiac examinations include transthoracic (TTE) and transesophageal echocardiography (TEE), 12-lead ECG and Holter monitoring for 24 hours. Routine TEE includes a search for right-to-left shunt (patent foramen ovale), atrial septal aneurysm and intracardiac thrombi. For research purposes the ascending aorta, the aortic arch and the proximal portion of the descending aorta is examined with respect to aortic plaques, defined as irregular thickening of the vessel wall of > 3 mm thickness [30].

#### *Blood tests*

Serum and plasma samples are taken for laboratory investigations including parameters of inflammation (e.g. hs-CRP, interleukin), lipid status and for future analyses of biomarkers that will emerge during the study. Biosamples are processed using standard operating procedures harmonized with the Munich biotech cluster m4 (http://www.m4.de).

#### *Stroke classification*

All initial strokes and recurrent events will be classified according to the TOAST and ASCO classification and both classification schemes will be used for the final analyses. The Classification is carried out by two experienced raters (ABK, FAW) who are blinded with regard to the results of carotid MRI. In case of discrepancy, a consensus will be reached by discussion.

#### *Carotid MRI*

All subjects are imaged twice (baseline and 12 month follow up) using a multi-sequence protocol [31] (Time-of-flight MR angiography (TOF-MRA), axial pre- and post- contrast black-blood T1-, PD- and T2- weighted sequences with fat suppression; best in-plane resolution 0.5 × 0.5 mm^2^) at 3 Tesla (Siemens Verio, Siemens Healthcare, Erlangen, Germany) with a dedicated 4-channel surface coil (Machnet B.V., Eelde, the Netherlands). Parallel imaging is used for all sequences with a parallel acquisition technique (PAT) acceleration factor of 2 for T1-, PD- and T2- weighted sequences. The protocol results in a total imaging time of 22:43 minutes for the carotid MRI. Gadolinium-DTPA-BMA (Gadobutrol, Bayer Schering, Leverkusen, Germany) of 0.1 mmol/kg (0.1 ml/kg) is given at a rate of 3 ml/s. Post-contrast T1w imaging is performed approximately 5 minutes after intravenous injection of the contrast agent. Fat suppression is used for pre- and post-contrast T1w, PDw, and T2w images to reduce signals from subcutaneous and perivascular fat. Each scan covers 30 mm (2 mm slice thickness × 15 matched images across the 5 sequences).

#### Dynamic contrast-enhanced carotid MRI

In the subgroup of patients included at the coordinating center (LMU Munich), an additional dynamic contrast-enhanced (2D-Saturation-Recovery Spoiled Gradient Echo) MRI series of the carotid arteries is acquired over five minutes after contrast administration consisting of two representative axial slices with a sampling interval of 1.8 seconds each. Using a PAT factor of 4, best in-plane resolution is 0.625 x 0.625 mm^2^. This sequence is acquired between the pre- and post-contrast T1-weighted sequences. The aim of this sub-study is to visualize markers of plaque vulnerability, such as neovascularization and inflammation [32] and to test whether increased plaque vascularization is associated with an increased progression rate and with a higher rate of stroke recurrence.

#### 18-Fluor-deoxyglucose positron emission tomography - MRI (18 F-FDG PET/MRI)

For a subgroup of patients recruited at the Technical University of Munich a novel, fully integrated PET/MR system (Biograph mMR, Siemens Healthcare, Erlangen, Germany) is used for the baseline scan. In addition to full 3 T-MRI capabilities including the acquisition of the aforementioned morphologic MRI-sequences, this hybrid PET/MR system permits the simultaneous acquisition and co-registration of a PET-dataset. To investigate the potential increase in diagnostic information provided by quantitative measures for the uptake of 18 F-FDG, patients are administered approx. 5 MBq/kg body weight of 18 F-FDG in combination with 20 mg of furosemide via i.v. bolus injection 3 hours prior to the baseline scan. Simultaneous acquisition of MRI-sequences and PET data is performed in analogy to the protocols published for cardiac imaging [33]. This additional PET-scan was approved by the Federal Office for Radiation Protection (Bundesamt für Strahlenschutz, BfS). The aim of this sub-study is to quantify plaque inflammation and to correlate plaque inflammation as assessed by PET/MRI to plaque morphology as assessed by carotid MRI. Furthermore, it will be tested whether increased plaque inflammation is associated with an increased progression rate and with a higher rate of stroke recurrence.

#### Contrast-enhanced ultrasound

Contrast enhanced ultrasound (CEUS) with a microbubble based contrast agent (SonoVue®, Bracco, Italy) will be applied in a subset of patients without risk factors for ultrasound contrast agents. The aim of this sub-study is to visualize markers of plaque vulnerability, such as plaque ulceration or neovascularization in the intima and the plaque [34] and to test whether increased plaque vascularization by ultrasound is associated with an increased progression rate by carotid MRI. Plaque vascularization will be graded on a 4-point scale (0 = absent, 1 = little, 2 = moderate and 3 = strong). Furthermore, the origin of the neovasculature will be recorded (1 = from the vessel wall, 2 = from the lumen). In addition presence or absence of plaque ulceration will be recorded.

### Sample size

Based on a pilot study by Freilinger et al. [24], we estimated that 40% of ipsilateral carotid artery plaques in patients with a cryptogenic stroke are complicated AHA-LT VI plaques. Thus, in 100 patients with cryptogenic stroke the prevalence can be assessed with a precision of +/− 10%. Our unpublished pilot data indicate a prevalence of complicated AHA-LT VI plaques in patients with defined stroke etiology of about 10%. To determine this difference with a power of 80% and 5% level of significance, 32 patients of each group (cryptogenic and control group) would be sufficient. Assuming a prevalence of complicated AHA-LT VI plaques of 30% in the cryptogenic stroke group, 64 patients in each group are needed.

The recurrence rate of ischemic stroke associated with complicated AHA-LT VI plaques will be determined as the time period until the next ischemic event. Assuming a recurrence rate of ischemic events of 8% [35] within 12 months following the index stroke and a hazard ratio of AHA-LT VI plaques ≥ 5 [19,34,35], 300 patients will be enough to demonstrate a significant difference in event rates between patients with and without AHA-LT VI plaques.

Previous longitudinal studies, which demonstrated associations of intraplaque hemorrhage with accelerated plaque progression and with new surface disruption, were able to show those associations with much smaller sample sizes [21,36]. Thus 300 patients will provide sufficient power to detect effects of certain plaque features on plaque progression.

### Imaging and statistical analysis

**Figure 1:**
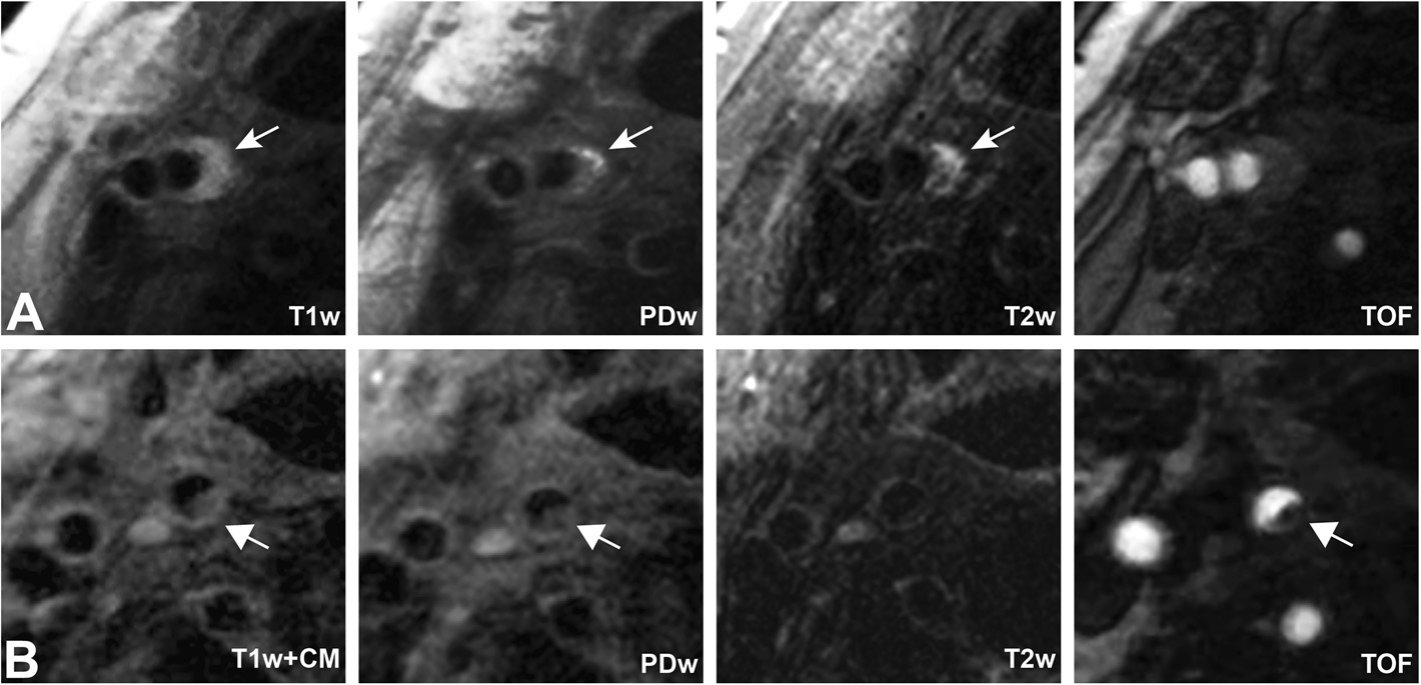
**Axial images of specific AHA-LT VI plaque features in a patient with acute ischemic infarct.** High-resolution black blood MRI of the carotid arteries demonstrate a large eccentric plaque with <50% stenosis in the right carotid artery, hyperintense on T1w, T2w and PDw sequences consistent with fresh intraplaque hemorrhage (**A**, arrow). Axial images 0.6 cm above the bifurcation show an area attached to the plaque, which is hypo- to isointense on T1w + CM, PDw and TOF, consistent with a mural thrombus (**B**, arrow). (T1w: T1-weighted black blood sequence, PDw: PD-weighted sequences, T2w: T2-weighted sequence, TOF: Time-of-flight MR angiography, T1w + CM: T1-weighted black blood sequence after contrast administration).

Atherosclerotic plaques in both carotid arteries (ipsilateral and contralateral to the ischemic stroke) will be recorded and classified according to the modified criteria of the American Heart Association [14]. Tissue components (necrotic core, calcification, hemorrhage, loose matrix) and type and location of hemorrhage will be identified and quantified based on previously published criteria [15,36]. For definition of a complicated AHA-LT-VI plaque, at least one of the following three criteria is required: fibrous cap rupture, intraplaque hemorrhage, juxtaluminal hemorrhage/mural thrombus (see Figure 1 for an example of a complicated AHA-LT VI). Area measurements of the lumen, outer wall, and tissue components are obtained using the custom-designed image analysis tool CASCADE (University of Washington, Seattle, US) [37].

All carotid and brain MRI examinations will be evaluated by two radiologists blinded to the clinical status. In case of discordant results, a consensus decision will be made. Image quality is rated on a five point scale (1 = worst, 5 = best), with MRIs ≥3 included for further analysis [31].

New ischemic lesions on brain MRI during follow up are defined as hyperintense lesions clearly visible on T2 weighted sequences.

All statistical analyses will be performed using SPSS and SAS. For determination of differences in prevalence of complicated AHA-LT VI plaques between patients with cryptogenic stroke and stroke with defined etiology the Chi square test will be used. Differences in the prevalence of complicated AHA-LT VI plaques ipsi- and contralateral to the ischemic stroke will be calculated using the McNemar Test. For the cross-sectional analysis the prevalence of AHA-LT VI plaques in patients with territorial infarcts, multiple embolic infarcts and small lacunar lesions will be compared. Furthermore, the difference in prevalence of AHA-LT VI plaques in patients with aortic arch plaques < 3 mm and > 3 mm will be determined. Data from the serial plaque study are analyzed in accordance to previous studies [38]. The effect of plaque morphology on cerebrovascular (TIA, amaurosis fugax and stroke) and cardiovascular events (myocardial infarction, vascular death) will be assessed using Cox proportional-hazards regression analysis. Cohen’s kappa test will be employed to evaluate the intra- and inter-reader agreement for identification of AHA-LT VI.

For distinctions of the stroke subtypes regarding recurrence rates of cerebrovascular events, Kaplan-Meier curves and Cox regression analysis will be used. For categorical variables, Fisher’s exact test and for continuous variables, an unpaired t-test will be applied. All p-values <0.05 will be considered as statistically significant.

## Discussion

This study differs from previous carotid plaque imaging studies in several ways. First, we focus on patients with cryptogenic stroke, classified by two raters and exclude patients with high grade stenosis (>70%), who usually undergo immediate invasive treatment and cannot be followed regarding plaque progression. Second, most previous studies have used a broader definition of the index event, including not only patients with a stroke documented by DWI-positive lesions on brain MRI, but also patients with TIA and amaurosis fugax [16,19], or even focused on asymptomatic patients [20,22]. Third, the time interval between the event and the MRI scan was up to 3 months [23], whereas CAPIAS includes patients within 7 days after symptom onset. Histopathological studies have shown that plaque features associated with vulnerability have the highest frequency in the first days after the ischemic event, thus suggesting that a short time period between event and MRI seems to be preferable [6,39]. Furthermore we excluded stroke patients younger than 50 years because the etiologic spectrum in this group differs from that in older subjects. The most frequent etiologic subgroups in the age range of 16–49 years are cardioembolism and arterial dissections while strokes due to large-artery atherosclerosis are extremely rare [40]. Finally, patients are systematically followed by clinical examination for 36 months and brain MRI for 12 months. Recent results of relatively small studies suggested that with improvement in best medical treatment the rate of recurrent events has decreased substantially in recent years. However, to date no larger trials are available which show the exact rate of recurrent events in ischemic stroke patients with <70% carotid artery stenosis and >2 mm atherosclerotic plaques and therefore it is impossible to provide precise estimates of the rate of stroke recurrence. In order to improve the power of the study we will use a clinical follow up period of 36 months and we will count silent ischemic lesions in the 12 months follow up brain MRI. Furthermore we will collect the number of TIA, amaurosis fugax and cardiovascular events.

This study also has limitations. Secondary prevention during follow up will differ among study participants depending on previous medication and stroke etiology. Patients with LAA or SVD are treated with antiplatelet agents, whereas patients with a cardioembolic etiology usually receive anticoagulants. Also, this study offers no information on the natural history of carotid plaques as all patients receive statin treatment for secondary stroke prevention. As demonstrated in several studies, statins influence the development of plaque burden, intraplaque hemorrhage and inflammation [39]. Another limitation is the additional scan time of the study-related MRI protocol, which may result in movement artifacts and severely affected stroke patients may refuse receiving an additional MRI.

So far, clinical and radiological baseline data of 79 patients have been analyzed. Stroke subtyping using the TOAST criteria showed the following distribution: 14% of subjects had LAA, 27% had CE, 13% had SVD, 5% had multiple potential causes (MPC) and 40% had cryptogenic stroke (Table 3). The small number of patients with LAA, the absence of other stroke causes and the high percentage of cryptogenic stroke result from our inclusion and exclusion criteria. Thus, the number of patients with LAA is smaller than expected as patients with ≥ 70% stenosis are excluded. Furthermore, we only include patients with advanced atherosclerotic disease, defined as plaques ≥ 2 mm in the carotid arteries, which might also explain the smaller than expected%-ages of patients with CE and SVD and the absence of patient with other stroke causes e.g. carotid artery dissection. Consistent with this notion, patients with cryptogenic stroke compromised almost half of our patient population, suggesting a potential link between atherosclerotic disease and the underlying cause of the ischemic stroke.

**Table 3:** Baseline characteristics of the first 79 patients enrolled in Munich

Age in years (median, Q1-Q3)		74 (68–83)
Female (n,%)		25 (32)
Systolic blood pressure at admission (mean, SD)		142 (19)
Diastolic blood pressure at admission (mean, SD)		78 (11)
BMI (mean, SD)		26 (3)
Pre stroke living condition (n,%)		
	At home alone	23 (32)
	At home with family or friends	46 (64)
	Institutionalized	2 (3)
NIHSS at admission (n,%)		
	0–4	51 (65)
	5–15	24 (30)
	≥16	4 (5)
mRS on admission (n,%)		
	0–2	47 (60)
	3-5	31 (40)
B I on admission (n,%)		
	0–19	5 (6)
	20–49	15 (19)
	50–74	6 (8)
	75–99	16 (20)
	100	36 (46)
MoCA (median, Q1-Q3)		26 (23–28)
Baseline medication (n,%)		
	Antiplatelet therapy	30 (38)
	Statin	30 (38)
	Oral anticoagulation	8 (10)
Ischemic Subtype (n,%)		
	Large artery atherosclerosis	11 (14)
	Cardioembolism	21 (27)
	Small vessel occlusion	10 (13)
	Other causes	0
	Multiple potential causes	4 (5)
	Cryptogenic	32 (40)
	Incomplete assessment	1(1)
Cardiovascular Risk Factors (n,%)		
	Coronary artery disease	17 (22)
	Peripheral artery disease	10 (13)
	Arterial hypertension	57 (72)
	Smoking	39 (49)
	Diabetes mellitus	17 (22)
	Hypercholesterolemia	22 (28)
	Atrial fibrillation	10 (13)

Data are given as medians (interquartile range), means (SD), and frequencies (percentages). BMI indicates body mass index; NIHSS, National Institute of Health Stroke Scale; mRS, modified Rankin Scale; BI, Barthel Index and MoCA, Montreal Cognitive Assessment.

An initial analysis of primary endpoint data showed a significantly higher prevalence of complicated AHA-LT VI plaques in cryptogenic stroke patients ipsilateral (37%) than contralateral (3%) to the ischemic stroke (p < 0.0001, McNemar). These preliminary results are in accordance with our pilot study suggesting a high prevalence of complicated AHA-LT VI plaques in the ipsilateral carotid artery in patients with cryptogenic stroke and unilateral anterior circulation infarcts. If confirmed in the entire cohort, our findings may have important implications for the understanding of stroke mechanisms. Moreover, this study might offer new diagnostic options by using high-resolution black-blood carotid MRI in the identification of risk patients for arterio-arterial embolism, as demonstrated in our first case report [41]. The question if these patients should receive a more aggressive or even interventional treatment has not been answered yet. Thus, we think that the results of our study could provide the basis for the planning of targeted interventional studies.

## Competing interests

The authors declare that they have no competing interests.

## Authors’contributions

ABK is responsible for the acquisition and analysis of clinical data and drafted the manuscript. FS is involved in acquisition and analysis of radiological data. FW has substantial input in acquisition of clinical data. HP contributed to the study protocol and is responsible for patient recruitment at TUM. TBB was responsible for the establishment of the radiological protocol and acquisition of radiological data at TUM. AB prepared the cardiac data assessment. KN had substantial input to the design of the study. CO revised the manuscript critically. TS is involved in acquisition and analysis of radiological data, designed the study protocol, and assisted in drafting the manuscript. MD designed the study protocol, and assisted in drafting the manuscript. All authors read and approved the final manuscript.

## Pre-publication history

The pre-publication history for this paper can be accessed here:

http://www.biomedcentral.com/1471-2377/13/201/prepub
